# Removal of cell surface heparan sulfate increases TACE activity and cleavage of ErbB4 receptor

**DOI:** 10.1186/1471-2121-10-5

**Published:** 2009-01-26

**Authors:** Jorma A Määttä, Kaisa Olli, Tiina Henttinen, Minna T Tuittila, Klaus Elenius, Markku Salmivirta

**Affiliations:** 1 Turku Center for Disease Modeling/Department of Cell Biology and Anatomy, University of Turku, Turku, Finland; 2 Turku Centre for Biotechnology, University of Turku and Åbo Akademi University, Turku, Finland; 3 Department of Medical Biochemistry and Molecular Biology and Medicity Research Laboratory, University of Turku, Turku, Finland

## Abstract

**Background:**

Nuclear localization of proteolytically formed intracellular fragment of ErbB4 receptor tyrosine kinase has been shown to promote cell survival, and nuclear localization of ErbB4 receptor has been described in human breast cancer. Tumor necrosis factor alpha converting enzyme (TACE) initiates the proteolytic cascade leading to ErbB4 intracellular domain formation. Interactions between matrix metalloproteases and heparan sulfate have been described, but the effect of cell surface heparan sulfate on TACE activity has not been previously described.

**Results:**

As indicated by immunodetection of increased ErbB4 intracellular domain formation and direct enzyme activity analysis, TACE activity was substantially amplified by enzymatic removal of cell surface heparan sulfate but not chondroitin sulfate.

**Conclusion:**

In this communication, we suggest a novel role for cell surface heparan sulfate. Removal of cell surface heparan sulfate led to increased formation of ErbB4 intracellular domain. As ErbB4 intracellular domain has previously been shown to promote cell survival this finding may indicate a novel mechanism how HS degradation active in tumor tissue may favor cell survival.

## Background

Heparan sulfate (HS) is a sulfated polysaccharide which consists of glucosamine and glucuronic or iduronic acid disaccharide units. Several HS chains are attached to a syndecan or glypican protein core. HS has been found to bind and regulate the activity of various extracellular matrix metalloproteases (MMP) such as MMP-1, MMP-2, MMP-7, MMP-9 and MMP-13 [1]. In Alzheimer's disease the activity of BACE1, an enzyme responsible for the production of the amyloidogenic peptide, has been shown to be directly regulated by interactions with HS [2].

Cell surface proteases take part in cell signaling by i) producing soluble extracellular mediators such as growth factors, chemokines and cytokines from membrane bound precursors [3] and ii) generating intracellular signaling molecules from transmembrane protein receptors [4,5]. One such cell surface protease is tumor necrosis factor alpha (TNF-α) converting enzyme, TACE [3].

TACE has also been shown to cleave various cell surface receptors including ErbB4 [6]. ErbB4 receptor belongs to the EGF receptor family of receptor tyrosine kinases (RTKs), which share homology with the avian erythroblastosis virus oncogenic factor v-erbB. It has been shown to stimulate cell survival, proliferation and differentiation [7,8].

Four different ErbB4 isoforms can be generated by alternative splicing [9,10]. The juxtamembrane isoform JM-a is recognized and cleaved by TACE, whereas JM-b is not cleavable [9]. The cytoplasmic isoforms either bind (JM-a CYT-1, JM-b CYT-1) or can not bind (JM-a CYT-2, JM-b CYT-2 phosphoinositide 3-kinase (PI 3-K) [10]. Proteolytically produced ErbB4 CYT-2 80 kDa fragments has been shown to favor cell survival [8].

The expression of heparan sulfate cleaving β-endoglucuronidase, heparanase, is tightly controlled in normal tissues [11,12]. However, in inflammed or cancer tissue and in several cancer cell lines the expression of heparanase has been shown to be elevated [13-15] and the high expression of heparanase has been linked to highly invasive cancers [15-17]. In breast cancer nuclear localization of ErbB4 receptor has been demonstrated and nuclear ErbB4 expression has been shown to be associated with unfavorable disease prognosis when compared to membraneous ErbB4 expression [18].

In this communication we describe for the first time that removal of cell surface HS increases membrane bound ErbB4 80 kDa fragment formation by TACE-like activity. Further, removal of cell surface HS enhanced the capability of living cells to cleave synthetic TACE substrate peptide suggesting that cell surface HS regulates TACE activity.

## Methods

### Preparation of expression constructs

Hemagglutinin (HA) -tagged human TACE encoding vector [19] was a generous gift from Professor A. Ullrich, Max Planck Institute, Germany. HA-tagged ErbB4 JM-a and JM-b CYT-2 receptor constructs were generated as follows: Sequence encoding the aminoterminal part of the full length receptor [8] was joined in front of a sequence encoding HA-tag at the C-terminus of ErbB4 CYT2 80 kDa fragment [20]. Flag-tagged syndecan-4 expression construct in pcDNA3.1 Neo vector (Promega, USA) was generated by trimming the flag-peptide encoding sequence to the 3'-terminus of syndecan-4 cDNA derived from MCF-7 cells.

### Cell culture

Generation and maintenance of the MCF-7 human breast cancer cells expressing human ErbB4 JM-a CYT-2 has been described previously [8]. T47D cells were maintained in RPMI-1640 medium supplemented with 10 FCS and glutamine. For immunoblot analysis cells were grown to 40–50% confluency on 6-well tissue culture plates. For confocal microscopy, cells were grown on coverslips in flat-bottomed 24-well tissue culture plates. Transfections were done by using Fugene-6 transfection reagent (Roche, Switzerland).

### Preparation and analysis of cell lysates

Cells grown to 70% confluency on 6-well tissue culture plates were washed twice with PBS prior to treatments. Incubations with enzymes and control treatments were performed at +37°C for 30 min in PBS supplemented with 10 μM calcium acetate and 10 mM glucose. Heparitinase and chondroitinase (Seikagaku, Japan) were used at 0.01 U/ml. After incubation cells were lysed as described previously [8] in the presence of TACE inhibitor 2 mM 1,10 orto-phenanthroline (Sigma-Aldrich, USA) [21]. For immunoprecipitation the NaCl concentration of the lysates were adjusted to 150 mM. The precipitations and immunoblots were performed as previously described [8].

### Measurement of TACE activity

Recombinant human TACE and TACE substrate peptide (Fluorescent substrate peptide III) were purchased from R&D Systems (USA). The amount of generated fluorescence was measured according to manufacturer's instructions after 60 min incubation at +37°C in the presence or absence of increasing concentrations of bovine lung heparin (Sigma-Aldrich, USA) or bovine kidney HS (Sigma-Aldrich, USA). TACE activity on living MCF-7 cells was measured after cells were grown to 80% confluency on flat-bottomed 96-well plates. Cells were incubated for 30 min in PBS containing 10 mM glucose in the presence or absence of 0.001 U/ml heparitinase or chondroitinase (Seikagaku, Japan). After incubation, cells were washed and the generation of fluorescent end product was followed at 5 min intervals. The enzyme assay buffer was supplemented with 0.2 mM phenylmethyl sulphonium fluoride and 5 mM EDTA.

### Confocal microscopy

Cells grown on coverslips were fixed and permeabilized with ice-cold methanol. Primary antibodies were used as following dilutions: HFR-1 monoclonal mouse anti-ErbB4 intracellular fragment (Neomarkers, USA) at 1:50; monoclonal rat anti-hemagglutinin 12CA5 epitope and monoclonal mouse anti-myc 9E10 epitope at 1:100 (Zymed, USA). Secondary antibodies were diluted in 10% FBS-PBS as follows: Alexa-568 goat anti-rat at 1:400; Alexa-488 goat anti-mouse at 1:400 (Molecular Probes, USA). Coverslips were embedded on Vecta Shield Hard Set mounting solution containing DAPI. Samples were analyzed with Zeiss LSM-510 Meta confocal microscope.

## Results and Discussion

**Figure 1:**
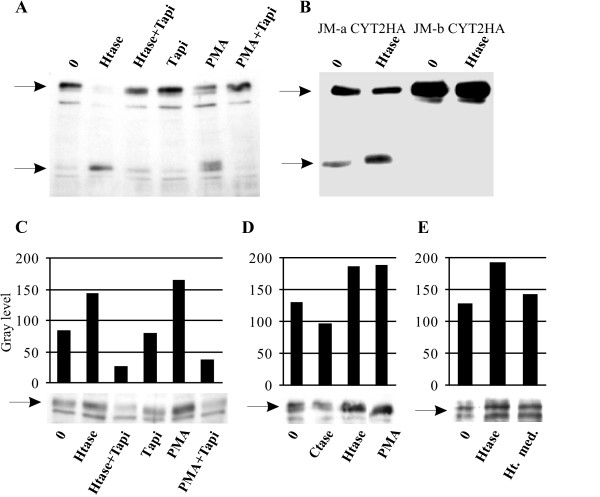
**A) The effects of heparitinase and PMA can be reverted by adding 40 μM TAPI-0 to the incubation medium**. MCF-7 cells were treated for 30 minutes and Triton-X100 soluble lysates containing 40 μg protein were subjected to immunoblot. The position of full length ErbB4 protein and ErbB4 80 kDa fragment as detected by the polyclonal sc-283 anti ErbB4 antibody are indicated by arrows. B) The effect of heparitinase is specific for TACE-cleavable (JM-a) ErbB4 isoform. MCF-7 cells were transiently transfected with ErbB4 JM-a CYT2HA or JM-b CYT2HA gene construct. Lysates were subjected to immunoblot with HA-specific monoclonal antibody. C) The effect of heparitinase treatment could be demonstrated in T47D cells treated similarly to MCF-7 cells. D) Degradation of chondroitin sulfate did not increase the formation of ErbB4 80 kDa fragment in T47D cells. E) Heat inactivated incubation medium from heparitinase treatment of T47D cells had only small effect on ErbB4 80 kDa fragment formation. The intensity of the ErbB4 80 kDa fragment staining as indicated in C and D was quantified with ImageJ software vs. 1.38 (NIH, USA). Beta-actin was used as load control (not shown). Abbreviations: Htase, heparitinase; Ctase, chondroitinase, PMA, phorbol myristyl acetate. Ht med. heat inactivated heparitinase incubation medium. The images are representative of at least three independent analyses.

Cleavage of ErbB4 receptor to soluble ectodomain and membrane-bound 80 kDa fragment can be induced by receptor-ligand binding and phorbol myristyl acetate (PMA) [22] and the cleavage is dependent on TACE activity [6,8]. The quantity of ErbB4 JM-a CYT-2 80 kDa fragment upon treatment of MCF-7 cells with heparitinase was increased to extent comparable to control treatment with PMA (Fig. 1A). No increase in the amount of ErbB4 80 kDa fragment could be detected after degradation of cell surface HS with the presence of TAPI-0, a potent inhibitor for TACE [8]. To ascertain that presence of TACE-cleavable ErbB4 juxtamembrane domain is required for the cleavage of the receptor induced by HS removal, MCF-7 cells were transiently transfected with carboxy-terminally hemagglutinin-tagged ErbB4 JM-a CYT-2 or JM-b CYT-2 receptor isoform. The carboxyterminal HA-tag has recently been shown not to affect the ErbB4 receptor cleavage [23]. As expected, no 80 kDa HA-immunoreactive protein was present in JM-b CYT2HA expressing cells (Fig. 1B). The enhancement of ErbB4 80 kDa fragment formation could be demonstrated also in the context of endogenously expressed ErbB4 in T47D human breast cancer cells (Fig. 1C). Degradation of chondroitin sulfate did not impose similar effect on ErbB4 80 kDa fragment formation as heparitinase (Fig. 1D). Degradation of cell surface HS can potentially release growth factors or induce their processing form membrane bound precursors which in turn may trigger growth factor receptor processing. Members of the epidermal growth factor family are generally heat-stabile [24] whereas heparitinase is readily inactivated in temperatures over 52°C [25]. Incubation supernatant from heparitinase treatment of T47D cells was heat inactivated 10 minutes at 65 °C and used to treat T47D cells. Only minor effect on ErbB4 80 kDa fragment formation could be demonstrated with heat-inactivated supernatant compared to heparitinase treatment (Fig. 1E). This indicated that the effect of heparitinase treatment appears to be mainly due to the removal of HS *per se*. However, growth factor release induced receptor processing may simultaneously happen in smaller extent.

**Figure 2:**
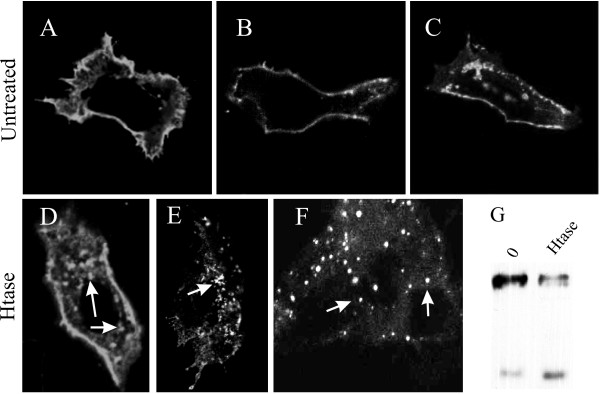
**Confocal microscopy illustration of samples of MCF-7 cells A-C) without treatment; D-F) treated 30 minutes with heparitinase (in figure F a group of three cells), Cells were stained with ErbB4 carboxy-terminus specific HFR-1 antibody**. Accumulation of immunoreactive perinuclear granules is indicated by arrows. G) Activity of Heparitinase was controlled by simultaneous Western analysis of ErbB4 cleavage from parallel samples.

Confocal immunofluorescence microscopy (Fig. 2) revealed that in untreated cells, the ErbB4 immunoreactivity was mostly detected at the plasma membrane with some perinuclear immunoreactive granules present, whereas heparitinase treatment increased the presence of perinuclear ErbB4 C-terminus-specific granules in MCF-7 cells.

**Figure 3:**
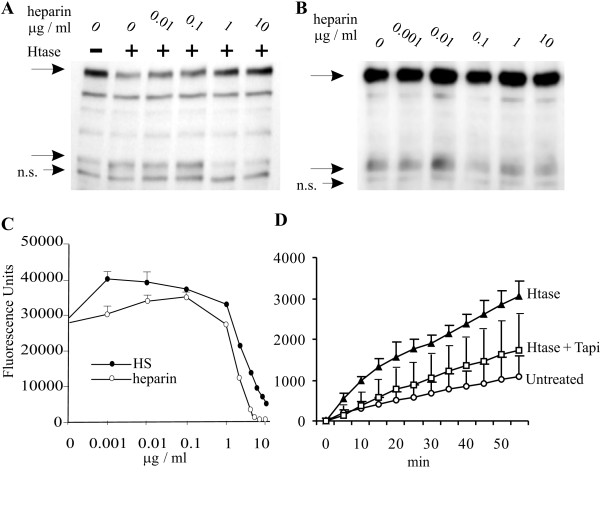
**Exogeneous heparin or HS inhibit heparitinase-induced ErbB4 80 kDa fragment formation**. A) Small concentrations of heparin slightly enhance the heparitinase-induced ErbB4 80 kDa fragment formation, whereas 1 μg/ml heparin and higher concentrations inhibit ErbB4 80 kDa fragment formation as indicated in lysates of T47D cells. B) Incubation with increasing concentrations of bovine lung heparin only did not have marked effect on cells. N. s., non-specific staining in A and B. C) Both heparin and heparan sulfate inhibited activity of recombinant TACE at high concentrations but displayed some enhancement of enzyme activity at low concentrations D) Heparitinase treatment of living MCF-7 cells enhanced cleavage of fluorescent TACE substrate peptide (p = 6 × 10^-11^) and the effect of heparitinase could be largely reverted by adding 40 μM TAPI-0 to the incubation medium (p = 10^-5^). The p-values were calculated with two-tailed pairwise Student's test comparing all time points. The enzyme activity analysis was performed three times with similar results. The data shown represents results from a single assay.

HS may mediate its effect on ErbB4 cleavage by interactions between polysaccharide and TACE. If degradation of HS leads to elevated TACE-like activity, it should be possible to inhibit it with exogeneous heparin or HS. Indeed, the enhanced formation of ErbB4 80 kDa fragment by heparitinase could be inhibited with the presence of 1 μg/ml heparin (Fig. 3A). Figure 3B shows that, exogenous heparin had no effect on ErbB4 80 kDa fragment formation in untreated MCF-7 cells. The capability of heparin and HS to inhibit TACE activity was further demonstrated by using recombinant TACE and a TACE-specific peptide substrate as shown in Figure 3C. Further, we evaluated heparin dependent inhibition of TACE activity on living cells. TACE substrate peptide cleavage was accelerated when MCF-7 cells were treated with heparitinase prior addition of substrate peptide (Figure 3D). Moreover, the effect of heparitinase could be largely reverted by addition of TACE inhibitor TAPI-0 to the enzyme assay buffer.

**Figure 4:**
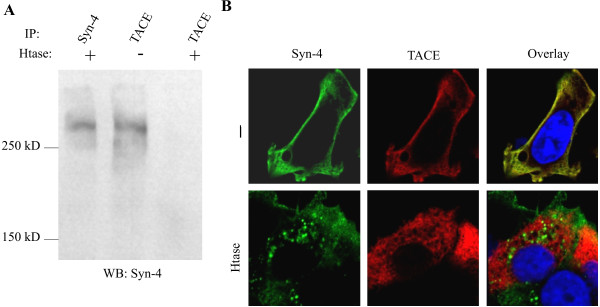
**A) TACE antibody was shown immunoprecipitate Syndecan-4 shown as high molecular weight smear in immunoblot by anti Syndecan-4**. The co-immunoprecipitation was abolished by heparitinase treatment. B) Syndecan-4 and TACE colocalize in MCF-7 cells. The colocalization is disrupted by heparitinase treatment.

Of the members of the syndecan proteoglycan family we could demonstrate co-immunoprecipitation and colocalization of syndecan-4 with TACE. Figure 4A shows that rat anti-HA antibody precipitates both the TACE-HA and syndecan-4-flag proteins from lysates of MCF-7 cells transiently transfected to express the tagged protein constructs. Further, the co-immunoprecipitation of syndecan-4 could be abolished by heparitinase treatment of the cells prior lysis. Confocal microscopy revealed that in untreated cells TACE is mostly located at the cell surface and the Golgi compartment. Also, syndecan-4 is mostly present at cell surface, and the cell surface immunoreactivity is located at the same structures as TACE-HA (Figure 4B). When cells were treated with heparitinase, syndecan-4 core protein translocated to intracellular vesicles.

Two mechanisms for HS-mediated regulation of TACE-activity can be postulated. HS may hinder TACE-mediated ErbB4 cleavage by forming steric barriers between the enzyme and its substrate cleavage site or directly inhibiting TACE activity with interactions between the enzyme and HS side chains. As soluble heparin and HS inhibited cleavage of fluorescent substrate peptide by recombinant TACE enzyme, the presence of the latter mechanism is suggested. This is further supported by the fact that exogenous heparin and HS hindered the HS degradation induced ErbB4 cleavage.

## Conclusion

In this communication removal of cell surface HS was shown to increase TACE activity and TACE-dependent formation of ErbB4 80 kDa intracellular domain. The high HS degrading activity reportedly present in tumor tissues [13-17] thus probably favors TACE-activity which may lead to elevated processing of ErbB4 and promotion of cell survival. Analogous mechanisms may be active also in inflammed tissue and may also concern other proteolytically processed transmembrane proteins.

## Abbreviations

Ctase: chondroitinase; CYT: cytoplasmic isoform; HA: hemagglutinin tag peptide; HS: heparan sulfate; Htase: heparitinase; JM: juxtamembrane isoform; TACE: tumor necrosis factor alpha converting enzyme

## Authors' contributions

JM, initiated the work hypothesis, planned and conduced experiments; KO performed part of confocal microscopy; TH participated in study design; MT prepared expression constructs; KE and MS supervised the work.
